# Dysregulation of iron homeostasis in airways associated with persistent preschool wheezing

**DOI:** 10.1186/s12931-023-02466-7

**Published:** 2023-06-23

**Authors:** Zhili Wang, Yu He, Qinyuan Li, Yan Zhao, Guangli Zhang, Zhengxiu Luo

**Affiliations:** 1 Department of Respiratory Medicine, Ministry of Education Key Laboratory of Child Development and Disorders, Chongqing Key Laboratory of Pediatrics, National Clinical Research Center for Child Health and Disorders, Children’s Hospital of Chongqing Medical University, Chongqing, 400014 China; 2 Department of Respiratory Medicine, Children’s Hospital of Chongqing Medical University, Chongqing, 401122 China

**Keywords:** Bronchoalveolar lavage, Children, Iron metabolism, Recurrent wheezing

## Abstract

**Background:**

Currently, there are no reliable clinical tools available to identify persistent asthma symptoms among preschool children with recurrent wheezing. We investigated iron homeostasis in the airways of preschoolers with recurrent wheezing and assessed whether iron homeostasis-related indices may reliably predict persistent wheezing.

**Methods:**

Iron levels and mRNA expression levels of iron homeostasis molecules were examined in bronchoalveolar lavage samples from 89 preschoolers with recurrent wheezing and 56 controls, with a 12-month follow-up conducted. Risk factors for persistent wheezing were identified using least absolute shrinkage and selection operator and multivariate logistic regression. The addition of predictive values of iron indices to the modified Asthma Predictive Index (mAPI) or clinical predictors was determined using area under receiver operating characteristic curves (AUC).

**Results:**

Preschoolers with recurrent wheezing had reduced iron levels in their airways, associated with significantly decreased expression of iron export molecule SLC40A1 and increased expression of iron intake factor TFR1 and iron storage factors FTH and FTL. Risk factors for persistent wheezing included mAPI positivity, iron predictors (lower expression of SLC40A1 and higher expression of FTL), and clinical predictors (aeroallergen sensitivity, shorter breastfeeding duration, and earlier age of first wheezing episode). The addition of information on iron predictors significantly enhanced the power of clinical predictors (AUC: 84%, increase of 12%) and mAPI (AUC: 81%, increase of 14%).

**Conclusions:**

Iron homeostasis is altered in the airways of preschoolers with recurrent wheezing. Adding information on iron-related indices to clinical information significantly improves accurate prediction of persistent wheezing in preschool-aged children.

**Supplementary Information:**

The online version contains supplementary material available at 10.1186/s12931-023-02466-7.

## Background

Wheezing is a common problem in preschool children and contributes to substantial morbidity, parental stress, and healthcare costs [1–3]. Recurrent wheezing is a common symptom of asthma during the preschool period (< 6 years); however, most children who experience wheezing episodes early in life do not go on to develop childhood asthma [4]. Early identification of preschool children with persistent wheezing or asthma is important to prevent morbidity associated with chronic asthma, such as long-term lung function decline [5, 6].

Various predictive tools, such as the modified Asthma Predictive Index (mAPI), have been developed based on readily obtainable clinical variables to identify young children at high risk of persistent wheezing or asthma [7, 8]. However, the sensitivity (ranging from 15 to 75%) and positive clinical predictive value (ranging from 12 to 74%) of these tools are low [7]. Recent studies have shown that combining clinical parameters and biomarkers, such as exhaled breath biomarkers and/or specific gene expression signatures, may improve prognosis prediction of preschool recurrent wheezing [9, 10]. Furthermore, accumulating evidence suggests that dysregulation of iron homeostasis is associated with childhood wheezing and asthma. Serum and exhaled breath condensate iron concentrations are significantly reduced in childhood asthma patients compared to healthy controls [11, 12]. A longitudinal study of mothers and children reported that lower umbilical cord iron status is associated with increased occurrence of wheezing [13]. However, it remains unclear whether iron homeostasis dysregulation is present in the airways of preschoolers with recurrent wheezing and whether iron homeostasis indices can add value to clinical parameters in prognosis prediction in preschool children with recurrent wheezing.

In the present study, we aimed to: (1) evaluate airway iron homeostasis in preschool children with recurrent wheezing by determining the expression levels of iron metabolism-related genes and iron levels in bronchoalveolar lavage (BAL) samples, and (2) investigate the value of adding iron homeostasis indices to clinical information in predicting persistent wheezing in preschoolers.

## Methods

### Patient recruitment

Patients were consecutively recruited between January 2021 and August 2021. Children aged 1–5 years who required a clinically indicated bronchoscopy with BAL collection for differential diagnostic purposes following recurrent wheezing were recruited for a single-center longitudinal study. Recurrent wheezing [14, 15] was defined as ≥ 3 episodes of physician-diagnosed wheezing treated with bronchodilators or corticosteroids. Children with recurrent wheezing and chronic disease (immunodeficiency, chronic lung disease, bronchopulmonary dysplasia, cystic fibrosis, bronchiolitis obliterans) were excluded. For comparison, a group of control children that underwent bronchoscopy with BAL collation for clinical indications (suspected foreign body, n = 28; chronic cough, n = 18; abnormal chest radiograph, n = 8; hemoptysis, n = 2) but without current wheezing, known sensitization to aeroallergens, or history of asthma or wheezing were also enrolled in the study.

### Processing of BAL samples

Bronchoscopy and collection of BAL samples were performed using standard procedures [16] with 0.9% sodium chloride warmed to room temperature. After collection, the BAL was gently aspirated and centrifuged at 3 000 rpm for 4 min at 4 °C, with the resulting supernatant stored at − 80 °C. The BAL cell pellet was resuspended in phosphate-buffered saline (PBS) and stored at − 80 °C.

### Wheezing phenotype

Episodic viral wheezing was defined as intermittent wheezing episodes, usually associated with viral respiratory tract infections, without wheezing between episodes. Multiple trigger wheezing was defined as wheezing observed during and between episodes, which is exacerbated by triggers such as allergens, exercise, crying, and laughing [17].

### Modified asthma predictive index

The mAPI was evaluated as positive if the child met at least one major (parental history of asthma, physician-diagnosed atopic dermatitis, and allergic sensitization to at least one aeroallergen) criterion or two minor (wheezing unrelated to colds, ≥ 4% eosinophilia, and allergic sensitization to milk, egg, or peanuts) criteria along with ≥ 4 wheezing episodes.

### Measurement of iron content in BAL supernatants

Iron content in BAL was measured using the chromogen method with an Iron Assay Kit (Sigma-Aldrich, USA) according to the manufacturer’s instructions [18]. BAL supernatants were mixed with the iron assay buffer. Standard and test samples containing iron reducer were mixed and incubated for 30 min at room temperature in the dark. Iron probe was added to each well and the mixture was incubated for 60 min at room temperature in the dark. Finally, absorbance was measured at 593 nm using a microplate reader.

### RNA extraction and measurement of iron metabolism-related gene expression

Total RNA was extracted from BAL cells using TRIzol reagent (Invitrogen, USA), and purified using a Micro Total RNA Extraction Kit (Jianshi Biotech, China). cDNA was synthesized using a PrimeScript RT Reagent Kit (TaKaRa, Japan). Reactions were carried out in a total volume of 10 μL, including 5 μL of TB Green®Premix Ex Taq™ II (TaKaRa, Japan), 0.2 μL of each specific primer, 2.6 μL of ddH_2_O, and 2 μL of cDNA. The relative expression levels of iron metabolism-related genes were calculated using the 2^−ΔΔCt^ method. GAPDH was used as an internal reference. Specific primers for each gene are provided in Additional file 1: Table S1.

### Patient follow-up and outcome measures

All participants with recurrent wheezing were followed up by phone calls to assess episodes of doctor-diagnosed wheezing at 6 and 12 months after discharge. The main outcome of persistent wheezing [19] was defined as two or more doctor-diagnosed wheezing episodes at 12 months post enrolment.

### Statistical analysis

All statistical analyses and visualizations were conducted using R software (v4.1.2; https://www.r-project.org/). Categorical variables were expressed as percentages. All continuous variables were summarized as medians (interquartile range [IQR]) based on their nonparametric distribution assessed by the Shapiro–Wilk test. We examined between-group differences in baseline characteristics, clinical presentation, laboratory data, and iron homeostasis indices using the Wilcoxon rank-sum, Pearson chi-square, or continuous correction chi-square tests, as appropriate. A *P-*value of < 0.05 was considered statistically significant.

We used least absolute shrinkage and selection operator (LASSO) regression [20] to quantify the contribution of all potential predictors (clinical plus iron homeostasis parameters, Additional file 1: Table S2) to identify important predictors and estimate their influence on persistent wheezing without overfitting the data. The “glmnet” [21] R package (v4.1.3) was used to fit LASSO regression, and tenfold cross-validation was used to select the penalty term lambda (λ). Potential risk factors selected by LASSO were subjected to backward stepwise logistic regression to identify the final clinical and iron-related predictors, with a threshold of *P* < 0.1 [22] considered significant. Backward stepwise logistic regression analysis was performed using the “MASS” [23] R package (v7.3.54).

As a reference, mAPI and clinical-related predictors were used to construct a mAPI model and clinical model, respectively. Thereafter, iron-related predictors were added to the two models separately to assess added predictive value. To determine the discriminative ability of the models (i.e., their ability to distinguish between patients with and without persistent wheezing), receiver operating characteristic (ROC) curve and area under the curve (AUC), also known as c-statistic [24], were calculated for the clinical and mAPI models separately as well as for iron predictors and combinations of iron predictors with the mAPI and clinical models. Discrimination was considered not better than chance at AUC of 0.5, moderate at AUC of 0.6 to 0.8, and good at AUC of greater than 0.8 [25]. DeLong’s test was used to assess the added value of iron predictors to the mAPI and clinical models. Differences were significant at *P* < 0.05.

The combination of predictors with the greatest discriminatory capacity was selected as the final predictive model for persistent wheezing. To assess overall performance of the final model, we calculated the scaled Brier score [23], ranging from 0 to 1, with lower scores indicating better calibration. The calibration (how closely the predicted probability reflects actual risk) was tested using the Hosmer–Lemeshow test [24] (< 0.05 indicates poor agreement between predicted probability and actual outcome) and visualized with a calibration plot [24].

The final model for predicting persistent wheezing was internally validated using bootstrapping [26] with 100 replicates. In bootstrapping, random samples drawn with a replacement from the original dataset were the same size as the original data size. For each bootstrap sample, a backward stepwise logistic regression model was fitted. The AUC of each bootstrap model was then calculated for the bootstrap sample and the original data in each bootstrap replication. Differences in these values were averaged over all 100 bootstrap replicates to calculate the amount of optimism for the AUC of the original model, which was used to calculate an optimism-adjusted AUC.

## Results

### Recruitment and baseline characteristics

A total of 89 participants with recurrent wheezing and 56 controls were recruited over the same period. The characteristics of participants are presented in Table 1. Overall, 62.9% (n = 56) of wheezers had an episodic viral wheezing phenotype. mAPI was positive in 40.4% (n = 33) of wheezers. Compared to the controls, children with recurrent wheezing were more likely to have a family history of asthma (*P* = 0.004), allergic rhinitis (*P* < 0.001), history of eczema (*P* = 0.035), prenatal smoking exposure (*P* = 0.004), eosinophilia (*P* = 0.005), and history of allergy (*P* = 0.08).

**Table 1:** Characteristics of the study population at baseline

Characteristic	Control, N = 56	Resolution of wheezing, N = 36	Persistence of wheezing, N = 48	*P* value*	*P* value†
Sex (% male)	29 (51.8)	24 (66.7)	28 (58.3)	0.36	0.44
Age (month), median (IQR)	2.29 (1.2, 4.2)	3.39 (1.8, 4.5)	2.92 (1.6, 4.2)	0.70	1.00
Preterm (%)	4 (7.1)	6 (16.7)	8 (16.7)	0.13	1.00
Cesarian section (%)	20 (64.3)	19 (52.8)	23 (47.9)	0.14	0.66
Breastfeeding time (month), median (IQR)	8.0 (6.0, 12.0)	9.5 (6.0, 12.0)	6.0 (5.8, 10.0)	0.14	0.008
Family history of asthma (%)	5 (8.9)	7 (19.4)	16 (33.3)	0.004	0.16
Allergic rhinitis (%)	12 (21.4)	19 (52.8)	35 (72.9)	< 0.001	0.057
History of eczema (%)	22 (39.3)	19 (52.8)	29 (60.4)	0.035	0.48
Allergy history (%)	7 (12.5)	9 (25.0)	10 (20.8)	0.080	0.65
Prenatal smoking (%)	11 (20)	17 (47)	21 (44.8)	0.004	0.75
Pet ownership (%)	4 (7.1)	1 (2.8)	4 (8.3)	0.73	0.39
First wheezing episode age (month), median (IQR)	−	10 (4.0, 24.0)	8 (4.5, 13.5)	−	0.67
Past wheezing episodes ≥ 5 (%)	−	16 (44%)	34 (71%)	−	0.015
Phenotype of wheezing (% EVW)	−	23 (63.9)	29 (60.4)	−	0.75
Positive mAPI (%)	−	7 (19.4)	26 (54.2)	−	0.001
mAPI major criteria (%)					
Parental asthma	2 (3.6)	5 (13.9)	5 (10.4)	0.13	0.74
Atopic dermatitis	1 (1.8)	4 (11.1)	4 (8.3)	0.15	0.72
Aeroallergens allergy	−	11 (30.6)	26 (54.2)	−	0.031
mAPI minor criteria (%)					
Wheezing without colds	−	13 (36.1)	19 (39.6)	−	0.75
Food allergy	−	11 (30.6)	18 (37.5)	−	0.51
Eosinophilia (≥ 4%)	4 (7.1)	7 (19.4)	15 (31.2)	0.005	0.22

*P* values of statistical difference between groups using either Wilcoxon rank sum test, Pearson's chi-squared test or continuous correction chi-square test depending on the characteristics of the data

*EVW* episodic viral wheezing, *IQR* interquartile range, *mAPI* modified Asthma Predictive Index

*P values for all patients with recurrent wheezing (included five patients who were lost to follow-up) versus controls

^†^*P* values for recurrent wheezers experienced persistent wheezing versus those patients without persistent wheezing

### Iron homeostasis dysregulation in airways of children with recurrent wheezing

**Fig. 1:**
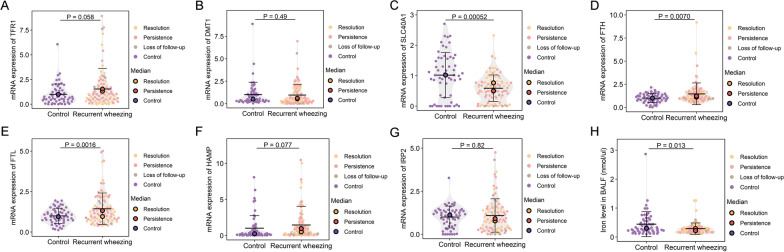
Altered expression levels of iron regulatory molecules in airways of preschool children with recurrent wheezing. mRNA expression levels of TFR1 **A**, DMT1 **B**, SLC40A1 **C**, FTH **D**, FTL **E**, HAMP **F**, and IRP2 **G** were assessed in BAL samples collected from preschool recurrent wheezers (n = 89) and controls (n = 56) using qRT-PCR. Iron levels in BAL supernatant collected from preschool recurrent wheezers (n = 89) and controls (n = 56) **H**. Black bars, mean ± standard deviation (SD); gray silhouette, probability density. Data from different groups are indicated by colored circles; medians are indicated by large circles. Statistical significance was assessed using Wilcoxon rank-sum test. *qRT-PCR* quantitative reverse transcription polymerase chain reaction

We assessed whether the expression of iron homeostasis molecules is altered in the airways of recurrent wheezing patients using quantitative reverse transcription polymerase chain reaction (qRT-PCR). We first assessed the mRNA expression levels of iron sequestration molecules transferrin receptor (TFR1) and ferrous ion membrane transport protein (DMT1) and found that TFR1 mRNA expression was increased (borderline *P* = 0.058) in the airways of recurrent wheezing patients compared to controls (Fig. 1A, B). We next assessed the expression of iron export, storage, and regulatory factors. Compared to the controls, preschoolers with recurrent wheezing showed significantly decreased expression of iron export molecule solute carrier family 40 member 1 (SLC40A1) (*P* < 0.001) and increased expression of iron storage molecules ferritin heavy chain (FTH) (*P* = 0.007) and ferritin light chain (FTL) (*P* = 0.0016) (Fig. 1C–E). In addition, expression of the iron regulatory molecule hepcidin antimicrobial peptide (HAMP) tended to be increased in patients with recurrent wheezing (*P* = 0.077) (Fig. 1F). There were no significant differences in the expression of iron sequestration molecule DMT1 (Fig. 1B) and iron regulatory protein 2 (IRP2) between the two groups (Fig. 1G).

We further examined iron levels in the airways of controls and recurrent wheezing patients. Iron levels in the BAL supernatants were reduced in the recurrent wheezing patients compared to the controls (*P* = 0.013) (Fig. 1H). Thus, results showed that the key factor associated with iron export (SLC40A1) was largely reduced, while factors associated with intracellular iron intake (TFR1) and storage (FTH and FTL) were increased in the airways of recurrent wheezing patients, which may explain the decrease in extracellular iron levels in the BAL supernatant.

### Follow-up assessment and determination of risk factors for persistent wheezing

In total, 84 participants (94.4%) with recurrent wheezing completed the 12-month follow-up. Of these, 48 (57%) experienced persistent wheezing. Patients with persistent wheezing had shorter breastfeeding time (*P* = 0.008), higher number of past wheezing episodes (≥ 5, *P* = 0.015), and higher positive rates of mAPI (*P* = 0.001) and aeroallergens (*P* = 0.031) compared to those patients without persistent wheezing (Table 1). Furthermore, the prevalence of allergic rhinitis tended to be higher in patients with persistent wheezing than in those without persistent symptoms (borderline *P* = 0.057, Table 1). In addition, iron levels (*P* = 0.031) and SLC40A1 expression (borderline *P* = 0.055) were lower, while FTL expression tended to be higher (*P* = 0.063) in participants had persistent wheezing (Additional file 1: Table S2). For other iron homeostasis-related molecules, no significant differences were found between the two groups.

**Fig. 2:**
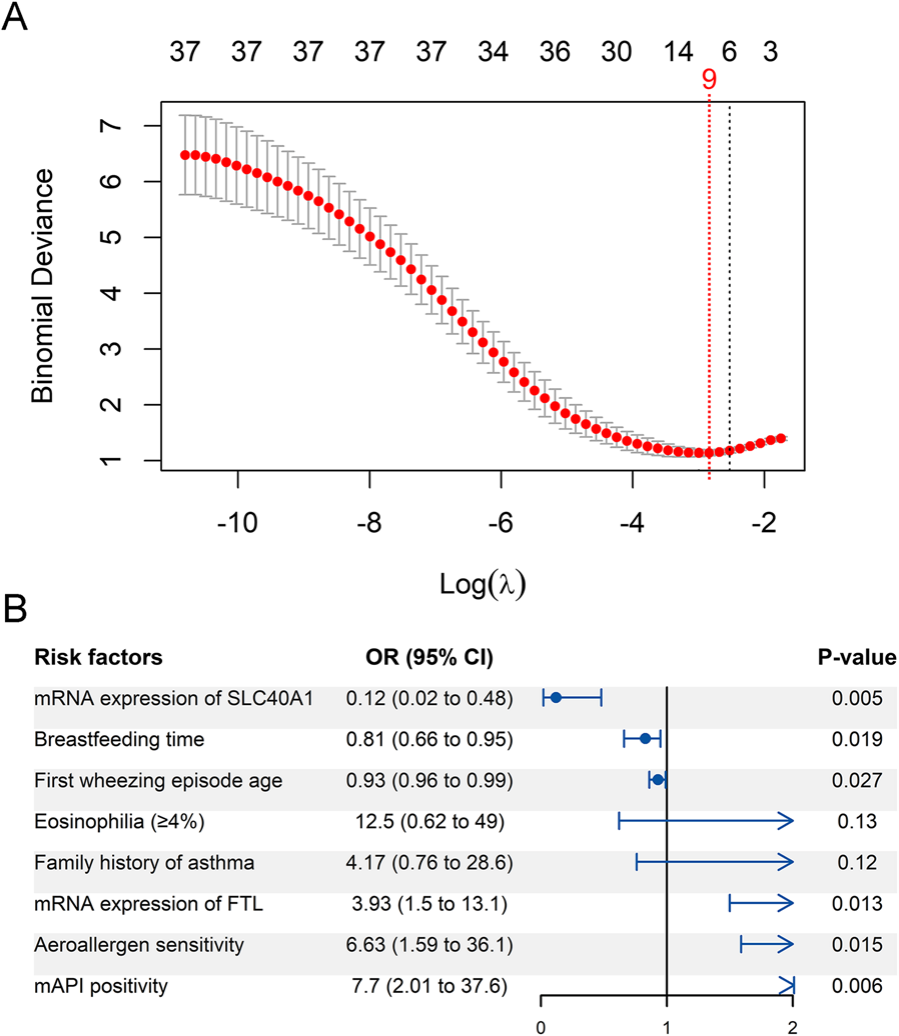
Determination of risk factors for persistent wheezing in preschool recurrent wheezers. Least absolute shrinkage and selection operator (LASSO) regression identified nine potential predictors for persistent wheezing according to minimal deviance criteria **A**. The partial likelihood deviance (binomial deviance) curve is presented versus log (lambda, λ). Red dashed vertical line represents minimum partial likelihood deviance. Forest plot shows backward stepwise logistic regression results for predicting persistent wheezing **B**. *CI* confidence interval; *mAPI* modified Asthma Predictive Index, *OR* odds ratio

We next investigated risk factors for persistent wheezing using LASSO regression and backward stepwise logistic regression. Of the 42 variables (Additional file 1: Table S2) used in variable selection, nine were retained by LASSO regression (Fig. 2A), the regression coefficients of which are provided in Additional file 1: Table S3. Backward logistic regression of these nine factors identified six risk factors (Fig. 2B) for persistent wheezing, including lower expression of SLC40A1 (odds ratio (OR): 0.12; 95% confidence interval (CI), 0.02–0.48; *P* = 0.005), mAPI positivity (OR: 7.7; 95% CI, 2.01–36.7; *P* = 0.006), shorter breastfeeding duration (OR: 0.81; 95% CI, 0.66–0.95; *P* = 0.019), earlier age of first wheezing episode (OR: 0.93; 95% CI, 0.96–0.99; *P* = 0.027), higher expression of FTL (OR: 3.93; 95% CI, 1.50–13.1; *P* = 0.013), and aeroallergen sensitivity (OR: 6.63; 95% CI, 1.59–36.1; *P* = 0.015). Lower mRNA expression of SLC40A1 was the strongest predictor of persistent wheezing.

### Predictive value of iron-related predictors for persistent wheezing

**Fig. 3:**
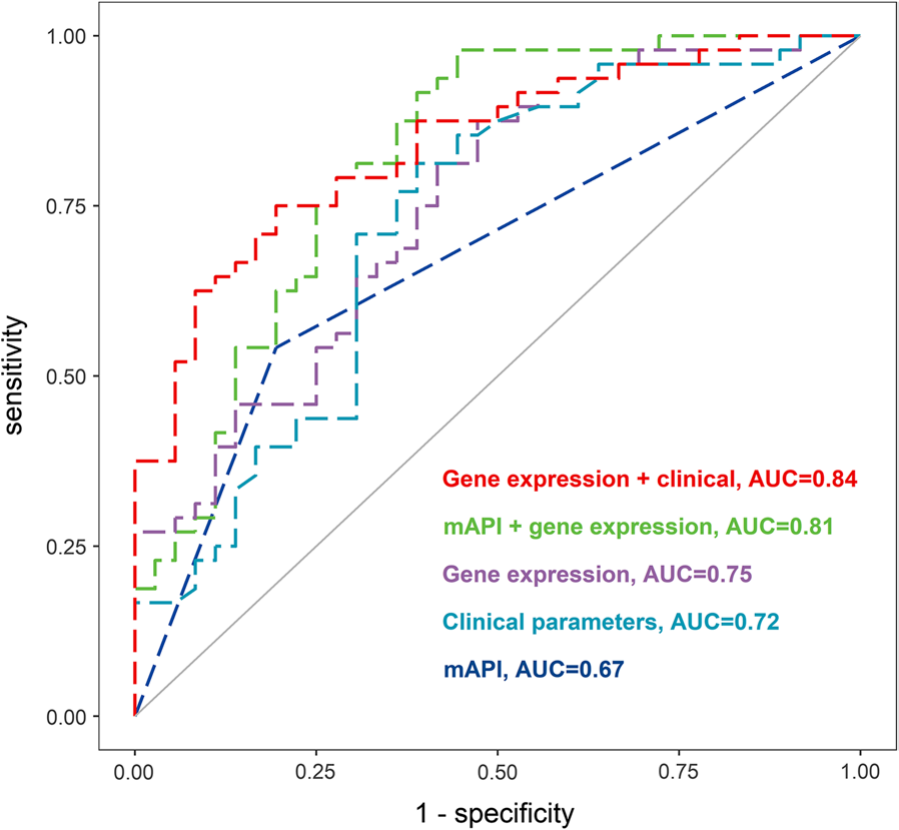
ROC curves for different models predicting persistent wheezing. Clinical parameters included breastfeeding duration, aeroallergen sensitivity, and age of first wheezing episode. Gene expression included expression of iron homeostasis-related genes (SLC40A1 and FTL). *AUC* area under the curve, *mAPI* modified Asthma Predictive Index, *ROC* receiver operating characteristic

We first assessed the predictive values of iron-related predictors, clinical model, and mAPI model by separately adding the iron-related predictors (SLC40A1 and FTL), clinical predictors (breastfeeding time, first wheezing episode age, and aeroallergen sensitivity), and mAPI into the logistic regression model. The overall predictive capabilities (AUC) of the iron-related predictors, clinical model, and mAPI model were 0.75, 0.72, and 0.67, respectively (Fig. 3, Table 2).

**Table 2:** ROC analysis of different models for the prediction of persistence of wheezing

Model	AUC (%) (CI)	*P* value	Sensitivity (CI)	Specificity (CI)	PPV (CI)	NPV (CI)
Iron + clinical predictors	0.84 (0.76–0.92)	< 0.001	0.75 (0.63–0.87)	0.81 (0.68–0.94)	0.84 (0.73–0.95)	0.71 (0.57–0.85)
Iron predictors + mAPI	0.82 (0.72–0.91)	< 0.001	0.98 (0.94–1.00)	0.56 (0.39–0.72)	0.75 (0.64–0.85)	0.95 (0.86–1.04)
Iron predictors	0.75 (0.65–0.86)	< 0.001	0.88 (0.78–0.97)	0.53 (0.37–0.69)	0.71 (0.60–0.83)	0.76 (0.59–0.93)
Clinical predictors	0.72 (0.61–0.84)	< 0.001	0.81 (0.70,0.92)	0.61 (0.45–0.77)	0.74 (0.62–0.86)	0.71 (0.55–0.87)
mAPI	0.67 (0.58–0.78)	0.001	0.54 (0.40–0.68)	0.81 (0.68–0.94)	0.79 (0.65–0.93)	0.57 (0.43–0.71)

*AUC* area under the curve, *CI* 95% confidence interval, *mAPI* modified Asthma Predictive Index, *NPV* negative predictive value, *PPV* positive predictive value, *ROC* receiver operating characteristic

We next assessed whether combining iron predictors could enhance the predictive values of the mAPI and clinical models. Compared to the mAPI model alone, adding data from the two iron predictors to the mAPI model significantly improved the AUC by 14% (*P* = 0.0010; Fig. 3, Table 2). Likewise, the AUC significantly improved by 12% when iron predictor data were added to the clinical model (*P* = 0.020; Fig. 3, Table 2).

### Prediction model for persistent wheezing

**Fig. 4:**
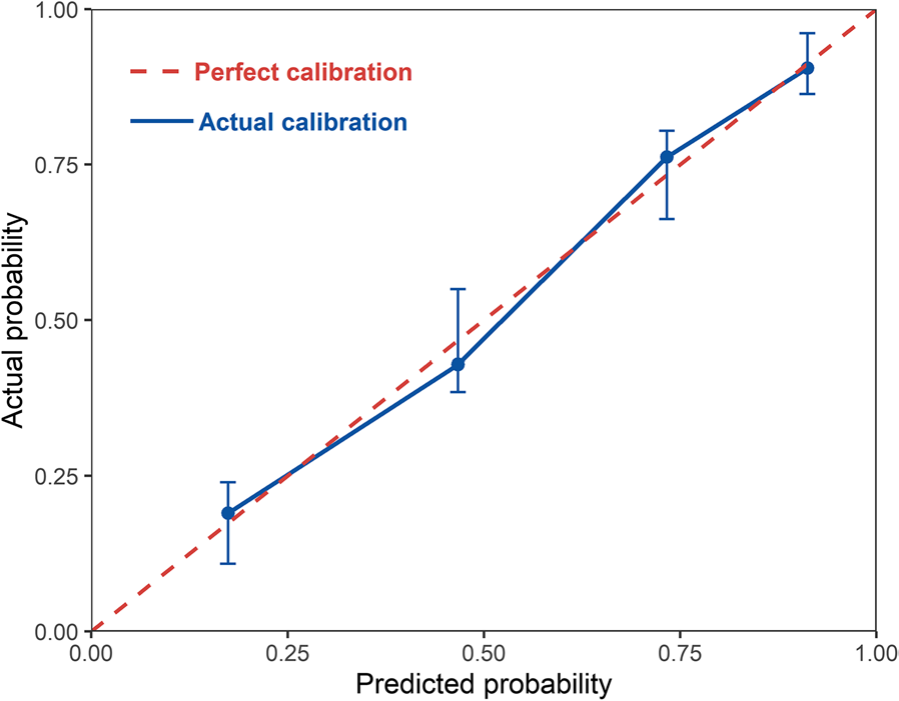
Calibration plot of final model for predicting persistent wheezing**.** All children with recurrent wheezing were grouped into quartiles according to their predicted probabilities. Average predicted probability for persistent wheezing among recurrent wheezers within each quartile was plotted against actual observed prevalence of persistent wheezing in that group. Red dashed line represents perfect calibration

The combination of iron and clinical predictors yielded the best predictive performance (AUC = 0.84, Fig. 3, Table 2). Therefore, the model combining two iron and three clinical predictors was selected as the final prediction model for persistent wheezing. Overall performance of the prediction model measured using the scaled Brier score was 0.14. The calibration plot (Fig. 4) showed good agreement between the predicted probabilities of persistent wheezing and observed frequencies, as confirmed using the Hosmer–Lemeshow test (*P* = 0.48). Finally, the prediction model was internally validated using bootstrapping with 100 replicates, which yielded an optimism-corrected AUC of 0.80, thus showing that the model well predicted persistent wheezing in preschooler with recurrent wheezing.

## Discussion

We investigated iron homeostasis in the airways of recurrent wheezing preschoolers and assessed the predictive value of iron-related predictors when added to mAPI or clinical predictors of persistent wheezing. Results suggested that the iron export molecule SLC40A1 was greatly reduced, while the iron intake factor TFR1 and iron storage factors FTH and FTL were increased in the airways of recurrent wheezing patients, consistent with the markedly lower iron levels in the BAL supernatants compared with the controls. Importantly, adding iron-related predictor data (mRNA expression of SLC40A1 and FTL) significantly improved the predictive ability of mAPI and clinical predictors of persistent wheezing, with the combined model of both iron and clinical predictors achieving the highest AUC (0.84).

We found that preschoolers with recurrent wheezing had reduced iron levels in their airways, significantly lower expression of SLC40A1, and higher expression of TFR1, FTH, and FTL. Most cells acquire iron by importing transferrin-bound iron from the blood via TFR1 [27], and ferritin, consisting of 24 heavy (FTH) and light (FTL) chain subunits, is the main iron storage protein [28]. Ferroportin (encoded by SLC40A1), is the only recognized mammalian iron exporter protein [27]. Thus, decreased expression of SLC40A1 and increased expression of TFR1, FTH, and FTL may explain the decrease in extracellular iron and possible accumulation of intracellular or tissue iron in the airways of patients with recurrent wheezing. Animal studies have demonstrated significant accumulation of iron in lung tissue in a house dust mite-induced murine asthma model, with pulmonary iron accumulation resulting in key features of asthma, including type 2 cytokine (interleukin-13) production, airway hyperresponsiveness, and airway fibrosis [29]. However, the precise role of iron metabolism disruption in the pathogenesis of recurrent wheezing is not yet clear, and thus further research is warranted.

ROC analysis showed that iron predictors alone had moderate predictive value for persistent wheezing (AUC = 0.75, Table 2). However, when iron-related predictors (SLC40A1 and FTL) were added to the mAPI or clinical predictors, the prediction of persistent wheezing was significantly improved. Although mAPI is the most commonly used index in predicting prognosis in preschoolers with recurrent wheezing [30], our results showed that mAPI alone performed poorly in predicting persistent wheezing (AUC = 0.67, Table 2), primarily due to its low sensitivity (0.54, Table 2), consistent with previous reports [14, 30, 31]. However, the combination of mAPI and iron predictors led to a substantial increase in sensitivity (0.98, Table 2) and predictive ability (AUC = 0.82, Table 2), while losing specificity (0.56, Table 2).

The present study indicated that shorter duration of breastfeeding, earlier age of first wheezing episode, and aeroallergen sensitivity were risk factors of persistent wheezing, consistent with previous studies showing that these factors are associated with the development of asthma in preschool-aged children with recurrent wheezing [8, 14, 32]. The model consisting of the three clinical predictors had a moderate predictive capacity for recurrent wheezing prognosis (AUC = 0.72, Table 2) but relatively poor specificity (0.61, Table 2). Interestingly, adding iron predictor information not only markedly increased the potential (AUC = 0.84, Table 2) to predict persistent wheezing but also increased the sensitivity (0.81, Table 2).

Our findings support previous studies showing that the addition of biomarkers and/or specific gene expression signatures to clinical parameters can help improve prognosis assessment in preschoolers with recurrent wheezing [9, 10]. Klaassen et al. [10] followed 202 preschoolers with recurrent wheezing and identified those who developed asthma at the age of 6 years. They found that adding information on nine exhaled volatile organic compounds and expression of six inflammatory genes to the asthma predictive index can significantly improve prediction (AUC = 0.95) of asthma in preschoolers with recurrent wheezing [10]. However, this model is somewhat complicated by the combination of multiple biomarkers and clinical parameters, making routine clinical screening and generalization in clinical practice difficult. More recently, in a longitudinal study of 135 preschool recurrent wheezers, Kreißl et al. [9] found that exhaled breath condensate pH combined with the asthma predictive index, serum allergen-specific immunoglobulin E (IgE), and several other clinical parameters can improve prediction (AUC = 0.94) for early detection of preschool recurrent wheezers with an increased risk of developing asthma. These and our findings highlight the need for integrated assessment of clinical and biomarker information and the need to develop more reliable prognostic biomarkers for young children with recurrent wheezing.

The present study has several limitations. First, the study population was restricted to hospitalized children and the measurements of iron-related indices were invasive and may be difficult to perform in the general pediatric population. Therefore, our results may have limited external validity to broader samples of children, such as outpatients. Additionally, the prognostic value of circulating iron parameters for preschool asthma patients as more accessible and less invasive indicators of iron metabolism deserves further exploration. Our results are also limited by the lack of long-term follow-up. Second, although we used a method to minimize overfitting and performed internal validation, this was a single-center study with a small sample size, and potential selection bias and unknown discrepancies among participants may have confounded study findings. External validation with independent samples is necessary to further evaluate the predictive performance of iron indices in other populations. Finally, while our preliminary study implicated iron metabolism dysregulation in the airways of preschoolers with recurrent wheezing and several iron-related indices as possible predictors of persistent wheezing, the underlying signaling pathways and exact roles of disordered iron metabolism in recurrent wheezing remain to be elucidated.

## Conclusions

Our results indicated that reduced iron levels in the airways of preschoolers with recurrent wheezing were associated with decreased expression of SLC40A1 and increased expression of TFR1, FTH, and FTL. The addition of iron-related indices (SLC40A1 and FTL) to clinical information enhanced the prognostic prediction of persistent wheezing. The model combining iron and clinical-related predictors achieved an AUC of 84%. However, further studies with more patients and longer follow-up are needed to validate these findings.

## Supplementary Information

**Additional file 1: Table S1.** Primer sequence for qRT-PCR analysis. **Table S2. **Characteristics of the preschoolers with recurrent wheezing. **Table S3. **The estimated coefficients of nine potential predictors for persistent wheezing selected by least absolute shrinkage and selection operatorregression.

## Data Availability

The datasets used and/or analyzed during the current study are available from the corresponding author on reasonable request.

## Abbreviations

AUC: Area under the curve
BAL: Bronchoalveolar lavage
BALF: Bronchoalveolar lavage fluid
DMT1: Ferrous ion membrane transport protein
FTH: Ferritin heavy chain
FTL: Ferritin light chain
HAMP: Hepcidin antimicrobial peptide
LASSO: Least absolute shrinkage and selection operator
IgE: Immunoglobulin E
IQR: Interquartile range
IRP2: Iron regulatory protein 2
mAPI: Modified Asthma Predictive Index
ROC: Receiver operating characteristic
PBS: Phosphate-buffered saline
qRT-PCR: Quantitative reverse transcription-polymerase chain reaction
SLC40A1: Solute carrier family 40 member 1
TFR1: Transferrin receptor
