# Supplementary material for: Dysregulation of iron homeostasis in airways associated with persistent preschool wheezing
Source: Respir Res. 2023 Jun 23;24:170. doi: 10.1186/s12931-023-02466-7 (PMC10290382; doi:10.1186/s12931-023-02466-7)
Supplement: Supplementary file 1 — Additional file 1: Table S1. Primer sequence for qRT-PCR analysis. Table S2. Characteristics of the preschoolers with recurrent wheezing. Table S3. The estimated coefficients of nine potential predictors for persistent wheezing selected by least absolute shrinkage and selection operatorregression. [file 12931_2023_2466_MOESM1_ESM.doc]

# Additional file 1

**Table S1.** Primer sequence for qRT-PCR analysis

| Gene | Primers | Sequence (5’→3’) |
| --- | --- | --- |
| SLC40A1 | Forward primer | TGGATGGGTTCTCACTTCCTG |
|  | Reverse primer | GTCAATCCTTCGTATTGTGGCAT |
| TFR1 | Forward primer | GGCTACTTGGGCTATTGTAAAGG |
|  | Reverse primer | CAGTTTCTCCGACAACTTTCTCT |
| DMT1 | Forward primer | ATCGGCTCAGACATGCAAGAA |
|  | Reverse primer | TTCCGCAAGCCATATTTGTCC |
| FTL | Forward primer | CCCCCATCTCTGTGACTTCC |
|  | Reverse primer | TCGTGCTTGAGAGTGAGCCT |
| FTH | Forward primer | TCCTACGTTTACCTGTCCATGT |
|  | Reverse primer | GTTTGTGCAGTTCCAGTAGTGA |
| IRP2 | Forward primer | GGCAGAAATCGAGAGAGGCTT |
|  | Reverse primer | TGTGTGAATCTGTGCCGACT |
| HAMP | Forward primer | CCCACAACAGACGGGACAAC |
|  | Reverse primer | TTTGATCGATGACAGCAGCC |
| GAPDH | Forward primer | GTCTCCTCTGACTTCAACAGCG |
|  | Reverse primer | ACCACCCTGTTGCTGTAGCCAA |

qRT-PCR, quantitative reverse transcription-polymerase chain reaction.

**Table S2.** Characteristics of the preschoolers with recurrent wheezing (all potential predictors considered in the LASSO regression).

| **Characteristic** | **Resolution of wheezing,**  **N = 36** | **Persistence of wheezing,**  **N = 48** | ***p* value***** |
| --- | --- | --- | --- |
| Sex (% male) | 24 (66.7) | 28 (58.3) | 0.44 |
| Age (month), median (IQR) | 3.39 (1.8, 4.5) | 2.92 (1.6, 4.2) | 1.00 |
| Preterm (%) | 6 (16.7) | 8 (16.7) | 1.00 |
| Cesarian section (%) | 19 (52.8) | 23 (47.9) | 0.66 |
| Breastfeeding time (month), median (IQR) | 9.5 (6.0, 12.0) | 6.0 (5.8, 10.0) | 0.008 |
| Family history of asthma (%) | 7 (19.4) | 16 (33.3) | 0.16 |
| Allergic rhinitis (%) | 19 (52.8) | 35 (72.9) | 0.057 |
| History of eczema (%) | 19 (52.8) | 29 (60.4) | 0.48 |
| Allergy history (%) | 9 (25.0) | 10 (20.8) | 0.65 |
| Prenatal smoking (%) | 17 (47) | 21 (44.8) | 0.75 |
| Pet ownership (%) | 1 (2.8) | 4 (8.3) | 0.39 |
| First wheezing episode age (month), median (IQR) | 10 (4.0, 24.0) | 8 (4.5, 13.5) | 0.67 |
| Past wheezing episodes ≥ 5 (%) | 16 (44%) | 34 (71%) | 0.015 |
| Phenotype of wheezing (% EVW) | 23 (63.9) | 29 (60.4) | 0.75 |
| Positive mAPI (%) | 7 (19.4) | 26 (54.2) | 0.001 |
| mAPI major criteria (%) |  |  |  |
| Parental asthma | 5 (13.9) | 5 (10.4) | 0.74 |
| Atopic dermatitis | 4 (11.1) | 4 (8.3) | 0.72 |
| Aeroallergens allergy | 11 (30.6) | 26 (54.2) | 0.031 |
| mAPI minor criteria (%) |  |  |  |
| Wheezing without colds | 13 (36.1) | 19 (39.6) | 0.75 |
| Food allergy | 11 (30.6) | 18 (37.5) | 0.51 |
| Eosinophilia (≥ 4%) | 7 (19.4) | 15 (31.2) | 0.22 |
| Atopy (%) | 20 (56%) | 33 (69%) | 0.21 |
| White blood cell count (×109/L) | 8.8 (7.5, 9.8) | 9.0 (6.9, 11.6) | 0.52 |
| Anemia (%) | 4 (11%) | 3 (6.2%) | 0.46 |
| Platelet counts (×109/L), median (IQR) | 360 (281, 456) | 358 (284, 450) | 0.65 |
| C-reactive protein (mg/L), median (IQR) | 0 (0, 0) | 0 (0, 0) | 0.65 |
| Procalcitonin (ng/mL), median (IQR) | 0.07 (0.04, 0.12) | 0.07 (0.03, 0.08) | 0.12 |
| Alanine aminotransferase (IU/L), median (IQR) | 21 (15, 25) | 20 (15, 23) | 0.69 |
| Aspartate aminotransferase (IU/L), median (IQR) | 38 (32, 43) | 37 (33, 41) | 0.85 |
| Serum albumin (g/L), median (IQR) | 45.9 (44.0, 47.9) | 46.5 (43.0, 48.0) | 0.94 |
| BALF total cell counts (105/mL), median (IQR) | 1,477 (880, 2,632) | 1,270 (885, 2,818) | 0.86 |
| BALF lymphocytes (%) | 16 (7, 23) | 12 (5, 22) | 0.50 |
| BALF neutrophils (%) | 16 (8, 30) | 16 (6, 33) | 0.79 |
| BALF macrophages (%) | 27 (21, 46) | 34 (24, 45) | 0.34 |
| BALF epithelial cells (%) | 10 (5, 28) | 11 (4, 23) | 0.89 |
| BALF eosinophils (%) | 0 (0, 0) | 0 (0, 0) | 0.78 |
| mRNA expression of SLC40A1, median (IQR) | 0.76 (0.42, 0.99) | 0.50 (0.31, 0.74) | 0.055 |
| mRNA expression of TFR1, median (IQR) | 0.73 (0.17, 1.78) | 1.04 (0.58, 1.65) | 0.113 |
| mRNA expression of FTH, median (IQR) | 1.11 (0.80, 1.65) | 1.22 (0.91, 1.71) | 0.524 |
| mRNA expression of FTL, median (IQR) | 0.96 (0.71, 1.47) | 1.33 (0.91, 2.16) | 0.063 |
| mRNA expression of IRP2, median (IQR) | 0.78 (0.43, 1.40) | 0.91 (0.35, 1.54) | 0.84 |
| mRNA expression of HAMP, median (IQR) | 0.52 (0.19, 1.32) | 0.99 (0.18, 2.10) | 0.28 |
| mRNA expression of DMT1, median (IQR) | 0.55 (0.28, 1.30) | 0.61 (0.15, 1.57) | 0.92 |
| Iron level in BALF, median (IQR) | 0.29 (0.18, 0.40) | 0.22 (0.16, 0.29) | 0.031 |

**p* values of statistical difference between groups using either Wilcoxon rank sum test, Pearson’s chi-squared test or continuous correction chi-square test depending on the characteristics of the data. BALF, bronchoalveolar lavage fluid; EVW, episodic viral wheezing; IQR, interquartile range; LASSO, least absolute shrinkage and selection operator; mAPI, modified Asthma Predictive Index.

**Table S3.** The estimated coefficients of nine potential predictors for persistent wheezing selected by least absolute shrinkage and selection operator (LASSO) regression.

| Variables | Coefficients |
| --- | --- |
| Breastfeeding time | -0.078 |
| First wheezing episode age | -0.0077 |
| Family of asthma | 0.11 |
| Inhaled allergens | 0.39 |
| Eosinophil percentage | 0.48 |
| SLC40A1 | -1.03 |
| FTL | 0.49 |
| DMT1 | 0.010 |
| mAPI | 0.94 |

mAPI, modified Asthma Predictive Index.
